# 

**Published:** 1911-03-25

**Authors:** 


### CONTENTS.

| | PAGE |
|---|---|
| **The Future of the Metropolitan Hospital Sunday Fund** | 725 |
| **Stage Children** | 726 |
| **Annotations—** Syphilis and "606"—The Latest Fad—The Doctor's Top Hat | 727 |
| **Medical Opinion and Movement** | 728 |
| **Hospital Clinics—** Infective Arthritis.—II. By W. D'Oyly Grange, M.D. Edin. | 731 |
| **Surgery—** Primary Sarcoma of the Appendix | 733 |
| **Special Articles—** The Practice Market | 733 |
| **Home and Continental Spas—** III.—Buxton | 739 |
| **Toxicology—** An Unusual Source of Lead Poisoning | 742 |
| **The Royal Army Medical Corps Section—** Regimental Camp Sanitary Reports | 743 |
| **Motoring Notes—** Retreading of Tyres | 744 |
| **The General Practitioner's Column—** Some New Remedies in Use | 745 |
| **Parliament and Publications** | 746 |
| **News and Events of the Week** | 747 |
| **Editor's Letter-Box** | 748 |
| **The Week in the Courts** | 748 |
| **Obituary** | 748 |
| **Coming Events of the Week** | 748 |
| **The Week's Work—** The London Hospital—"Hospital Night" at the Theatres—University College Hospital Appeal—The Royal Infirmary, Edinburgh—A French Institute for Puericulture—A Want in Hospital Literature—A Comparison of Different Methods—This Week's Drug Market | 749 |
| **Special Institutional Articles—** I.—Public Hospitals and their Maintenance | 751 |
| Legacies to Hospitals | 753 |
| Stage Management at a Hospital | 754 |
| **Construction and Administration—** Water Supply | 755 |
| Electric Clocks for Hospitals | 755 |
| **University Education in London** | 756 |
| **Institutional Notes and News** | 757 |
| **The Hospital World** | 759 |
| **Jottings** | 759 |
| **Gifts and Bequests** | 760 |
| **Appointments Vacant** | 760 |
| **New Appliances &c.** | 760 |

